# *Borrelia* Infection in Bank Voles *Myodes glareolus* Is Associated With Specific DQB Haplotypes Which Affect Allelic Divergence Within Individuals

**DOI:** 10.3389/fimmu.2021.703025

**Published:** 2021-07-26

**Authors:** Kristin Scherman, Lars Råberg, Helena Westerdahl

**Affiliations:** ^1^ Molecular Ecology and Evolution Lab, Department of Biology, Lund University, Lund, Sweden; ^2^ Functional Zoology, Department of Biology, Lund University, Lund, Sweden

**Keywords:** major histocompatibility complex, DQB, *Borrelia*, bank vole, disease resistance

## Abstract

The high polymorphism of Major Histocompatibility Complex (MHC) genes is generally considered to be a result of pathogen-mediated balancing selection. Such selection may operate in the form of heterozygote advantage, and/or through specific MHC allele–pathogen interactions. Specific MHC allele–pathogen interactions may promote polymorphism *via* negative frequency-dependent selection (NFDS), or selection that varies in time and/or space because of variability in the composition of the pathogen community (fluctuating selection; FS). In addition, divergent allele advantage (DAA) may act on top of these forms of balancing selection, explaining the high sequence divergence between MHC alleles. DAA has primarily been thought of as an extension of heterozygote advantage. However, DAA could also work in concert with NFDS though this is yet to be tested explicitly. To evaluate the importance of DAA in pathogen-mediated balancing selection, we surveyed allelic polymorphism of MHC class II DQB genes in wild bank voles (*Myodes glareolus*) and tested for associations between DQB haplotypes and infection by *Borrelia afzelii*, a tick-transmitted bacterium causing Lyme disease in humans. We found two significant associations between DQB haplotypes and infection status: one haplotype was associated with lower risk of infection (resistance), while another was associated with higher risk of infection (susceptibility). Interestingly, allelic divergence within individuals was higher for voles with the resistance haplotype compared to other voles. In contrast, allelic divergence was lower for voles with the susceptibility haplotype than other voles. The pattern of higher allelic divergence in individuals with the resistance haplotype is consistent with NFDS favouring divergent alleles in a natural population, hence selection where DAA works in concert with NFDS.

## Introduction

Resistance to infectious disease depends on the detection and elimination of pathogens by the host immune system. Pathogens are first detected by various components of the innate immune system, which subsequently stimulate an acquired immune response where the Major Histocompatibility Complex (MHC) plays an important role. The class I and class II genes in the MHC encode molecules that present antigens to T-cells. An antigen presented by MHC, known as a peptide-MHC complex, will ultimately trigger a specific immune response. MHC class I presents antigens to cytotoxic T-cells, while MHC class II presents antigens to helper T-cells (1, 2). The MHC genes are famous for their allelic richness. There are typically 10 to 50 distinct alleles per polymorphic MHC gene (HLA A-C, DRB and DQB) in local human populations (3), whereas worldwide, thousands of different alleles have been recorded for the classical class I and class II genes (http://hla.alleles.org/nomenclature/stats.html). This pattern is thought to be similar in all vertebrates. How can such excessive allelic richness be maintained? Due to its crucial role in the immune system, pathogen-mediated balancing selection (PMBS) is generally invoked to explain MHC allelic richness. Three major hypotheses have been put forward: Heterozygote Advantage (HA), Negative Frequency Dependent Selection (NFDS) and Fluctuating Selection (FS). HA asserts that individuals that are heterozygous at MHC loci have increased fitness because they can present more antigens (4, 5). In NFDS, protective MHC alleles are prevented from going to fixation, because pathogens evolve genotype-specific escape variants to common alleles (6). FS maintains different MHC alleles because the composition of the pathogen community varies in time and/or space (7). HA, NFDS and FS are not mutually exclusive and it has proved challenging to establish their relative importance (8).

Empirical support for HA is comparatively rare (9–12), and HA is often found in parallel with the effect of specific alleles (13–16). There is a fair number of studies reporting associations between particular MHC alleles and infectious diseases in humans [reviewed in (17–19) and other animals (reviewed in (8, 10); e.g (14, 20–22)] consistent with NFDS/FS. However, associations between particular MHC alleles and infectious diseases in humans, and other animals (23), are sometimes inconsistent between studies and low sample sizes have been one explanation for such discrepancies. With more knowledge about the MHC region and the linkage of the genes therein it is now evident that chromosomal organization matters (19), hence analyses of effects of MHC on resistance/susceptibility to disease should preferably consider full MHC-haplotypes. Recent work in non-model organisms has shown that haplotype inference is feasible also in species with several to many paralogous genes (24–26).

MHC genes do not only have high allelic richness, but the alleles at a given locus often have high functional sequence divergence too. The Divergent Allele Advantage (DAA) hypothesis provides an evolutionary explanation to the extensive allelic divergence observed in the MHC genes. According to DAA, MHC alleles with little or no overlap in their peptide-binding repertoire will cover a wider range of antigenic space than MHC alleles with more similar binding properties (27, 28). Like in HA, heterozygous individuals have a fitness advantage compared to homozygotes, but in addition, heterozygotes with more dissimilar alleles (i.e. higher allelic divergence within individuals) have an advantage compared to heterozygotes with relatively similar alleles (28). Thus, DAA can be thought of as an extension of HA that adds variation among heterozygotes, i.e. selection for ability to recognize a wide range of antigens, predicting a positive correlation between allelic divergence within individuals and resistance. *In-silico* modelling of MHC class II loci have shown a significant positive effect of allelic divergence within individuals on ability to bind putative antigens from a variety of pathogens (28, 29). Empirically, allelic divergence within individuals has been shown to play a significant role in relation to intenstinal parasites (16, 30–32).

Apart from selection for allelic divergence within individuals, allelic divergence at the population level could also be a result of selection for specific MHC alleles with minimal overlap to other MHC alleles in the gene pool. Accordingly (27), speculated that resistance alleles favoured by NFDS are likely to be divergent from the common (susceptibility) alleles in the population. This will be the case if pathogens can escape MHC binding of a set of related alleles, but are more likely to be detected by MHC molecules with divergent binding properties. Under this scenario, selection will favour rare alleles that are divergent at the population level. If so, selection for divergent alleles at the population level may result in high allelic divergence within individuals for individuals carrying resistance alleles under NFDS.

In the present study, we test for DAA using data from a natural population of bank voles *Myodes glareolus* that have been screened for MHC class II DQB genotype and infection status with the tick-transmitted bacterium *Borrelia afzelii*. Each individual carries one to eight MHC class II DQB alleles, indicating that the DQB gene is present in up to four copies (33). MHC class II alleles are inherited in blocks (i.e. there is high linkage disequilibrium) and data from 527 bank voles, combined with data from 12 families, made it possible for us to infer DQB haplotypes. We set out to test if (1) infection status is associated with specific MHC haplotypes (indicating NFDS and/or FS), with MHC diversity, and/or with allelic divergence within individuals (DAA), and (2) if bank voles with resistance haplotypes have more divergent MHC genotypes than bank voles without such haplotypes.

## Methods

### Study Species and Sampling

*Myodes glaerolus* is one of the most abundant vertebrate species in northern Europe (34) and one of the main host reservoirs of the tick-transmitted bacterium *B. afzelii* (35). *Borrelia afzelii* spirochetes are transmitted from voles to the larval stage of *Ixodes ricinus* (sheep ticks). Tick larvae moult into nymphs which transmit the infection again to voles or to larger mammals including humans. *Borrelia afzelii* infections can reduce reproductive success of bank voles (36). Infections are more or less chronic in bank voles, which results in that prevalence increase with age. In our study population, approximately 25% of the adult individuals are infected (37).

We collected samples from live-trapped bank voles at Kalvs Mosse, east of Lund, southern Sweden (N 55 42.470’, E 13 29.216’), a site consisting of about 0.25 km^2^ deciduous wood land with dense undergrowth vegetation. Traps (Ugglan special, Grahn AB, Sweden) were baited with grains and apple and left over night. A targeted trapping grid with multiple transects of 20 traps were established in the first trapping session in June 2008 and returned to every sixth week, in early August, mid September, and late October the same year. Females that gave birth in the trap during night were brought to the animal facility at Stensoffa field station and kept until pups were independent, whereafter all were released at the site of trapping.

Bank voles weighing <15g were categorized as juveniles, voles between 15 and 20g as subadults and voles >20g as adults. At first capture, a microtransponder (Trovan ID-100B, AEG ID, Ulm, Germany) was inserted subcutaneously on the back to allow for identification upon recapture. In total, we obtained skin biopsies (ear punch samples) from 726 individuals (main data set collected in the field) during the four trapping sessions. 397 bank voles were recaptured on at least one occasion (34.2%). We also obtained samples from 14 families (family data; female and 3-6 pups per female). Samples were stored in 70% ethanol for later DNA extraction (35, 37). All animal procedures were approved by the Malmö/Lund ethical board for animal experiments (permits M101-06 and M141-10).

### DQB Amplicon Sequencing, Data Quality Control, and Haplotype Inference

We performed PCR with 454 fusion primers on 572 bank voles [randomly selected from the main data set collected in the field (726 individuals), from here and onwards called main data set]. The DQB specific primer set (MyglDQBfw and MyglDQBrv) amplify 205 out of 272bp in DQB exon 2 which is the most polymorphic exon and contains the majority of peptide binding residues. A subset of samples (N=14) were amplified in two independent PCR reactions to allow for evaluation of genotyping accuracy. PCR products were checked on agarose gels, and amplicons were pooled, purified and sequenced in four lanes of a single 454 FLX run at the DNA sequencing facility, department of Biology, Lund University. Sequences containing both forward and reverse primers, as well as complete tags (16fw tags and 16rv tags as described in (38), were extracted from the multifasta files and assigned to individuals using the software jMHC (39). As a first step we disregarded any sequences shorter than 200bp since our previous work did not find any indels (33). We also disregarded sequence variants with less than three reads in total across all samples, as well as all amplicons that contained fewer than 100 reads in total, before proceeding to further filtering. This restriction resulted in a loss of data from 21 bank voles.

As a second filtering step, we have previously removed sequence reads with a low per-amplicon frequency (33). The rationale behind this step of the data processing is based on the assumption that variants represented only rarely within a sample most likely appear as a result of sequencing errors (40, 41). While this is true, this step unfortunately disregard read numbers that actually contain important information about the true alleles (TAs). This is because the most common sequence errors are generated by either over or under-calling of identical base pairs relative to a real sequence (42). Following the same logic as in the Stepwise Threshold Clustering method (42), we therefore combined reads from artefact alleles (AAs) containing homopolymer insertion/deletion errors with reads from the more abundant TAs from which the AAs are derived (sequences differ only by homopolymer insertion/deletion and are found within the same amplicon), an approach that have been used to filter 454 MHC amplicon data in a wide range of species (26, 43). We identified TA-AA pairs by importing all unique sequences with more than 10 reads to Geneious 4.7.6., where we used the ‘Assemble’ functions to create alignments. Homopolymer errors could then be identified and manually corrected. Sequence reads were merged by the online tools ‘seqeqseq’ (http://mbio-serv2.mbioekol.lu.se/apps/seqeqseq.html) and ‘merge matrix’ (http://mbio-serv2.mbioekol.lu.se/apps/mergeMatrix.html). As acknowledged by (42), the process of combining the information of AAs with their TA templates increase the weight of evidence that the more frequent sequences in the data set are indeed TAs. This lowers the risk of false negatives in the next filtering step where low frequency variants are removed on a per individual basis.

Previous studies have used a stringent threshold of 3% to sort out AAs (33, 38, 40), that often occur at a lower frequency within individuals than TAs. The merging of reads from homopolymer errors with their original TAs, made it possible for us to keep sequences with more than 2.5% relative abundance per individual without increasing the risk of false positives. Sequences that met this 2.5% threshold in at least two amplicons (two independent PCRs should still be considered a gold standard in MHC genotyping), were identified with the web-application popMatrix (http://mbio-serv2.mbioekol.lu.se/apps/popMatrix.html). This last step has been shown to efficiently remove errors originating from PCR amplification such as chimeras (33). No chimeras, frame shifts or stop codons were found in the alleles that made it through all filtering steps.

Six new alleles (Mygl-DQB*41, DQB*70-74) were identified that had not been described in our previous study (33), five of which were not reported previously (GenBank accession numbers, BankIt2469430: Mygl-DQB*70: MZ359152, BankIt2469430: Mygl-DQB*71: MZ359153, BankIt2469430: Mygl-DQB*72: MZ359154, BankIt2476171: Mygl-DQB*73: MZ468899, BankIt2476171: Mygl-DQB*74: MZ468900). Thirteen out of 14 technical duplicates (independent PCRs) had more than 100 reads and could be used for validation. All 13 replicates matched to 100%.

To amplify exon 2 amplicons from 14 bank vole families (family data), we used the same DQB specific primer set as mentioned above, but the set-up of the PCR was tailored for Illumina MiSeq as described in (44). These PCR products were DNA sequenced using 300 bp paired-end Illumina MiSeq sequencing at the DNA sequencing facility, department of Biology, Lund University. After initial processing where the primer sequences were removed the raw reads were filtered as described for genomic DNA (gDNA) in (44). No chimeras, frame shifts or stop codons were found in the alleles that made it through all filtering steps.

MHC alleles are known to segregate in tightly linked haplotype blocks. Even if it is not possible to assign alleles to individual loci within MHC II DQB in bank voles at this point, we have been able to identify a limited number of combinations of highly associated alleles that most probably originate from separate loci on the same chromosome, i.e. haplotypes. To do this, we tested for associations between MHC alleles using cross-tabulation and Pearson’s correlations coefficient applied to binary data (Φ). Some allelic pairs always appeared together within individuals, but all pairwise relationships were not reciprocal. For example, the alleles DQB*03, DQB*04 and DQB*05 are always associated with each other (Φ=1) and with allele DQB*02, but not vice versa. By first identifying the individuals with perfectly associated allelic pairs, we were able to look for new associations in a step-wise manner. For example, by excluding individuals carrying the haplotype DQB*02*03*04*05, we found that all other bank voles carrying DQB*02 (i.e. not in combination with DQB*03, DQB*04 and DQB*05) also carried the alleles DQB*01, DQB*24 and DQB*31. Through this procedure we were able to infer nine out of the ten haplotypes which were found in >5% of the investigated individuals fully and the tenth haplotype (H12) partially (**Table 1** and **Table S1**).

**Table 1:** Allelic composition of the ten haplotypes (*Haplotype*, H01, H02 etc.) that occurred in >5% of the studied individuals.

Haplotype	Alleles	No. alleles	N	%
H01	*02*03*04*05	4	185	35.1
H02	*01*06*13	3	107	20.3
H04	*09*10	2	79	15.0
H05	*11*27	2	77	14.6
H06	*01*16	2	65	12.3
H08	*01*02*24*31	4	45	8.5
H09	*36*37*39	3	44	8.3
H10	*32*33	2	42	8.0
H11	*12*19	2	34	6.5
H12	*18*22	2	27	5.1

These ten haplotypes were also found in an independent data set with DQB genotypes from 12 families with known mother-offspring relations (**Table S1**). The composition of alleles (MyglDQB*01, *02 etc.) per haplotype (Alleles), number of different alleles per haplotype (No. alleles) and the frequency of the haplotype out of 527 individuals (N and %).

We validated our inference of haplotypes with data obtained from an independent data set with DQB genotypes from mother and offspring from 12 families, the mother was not genotyped in two of the 14 families. This data allowed us to directly track haplotype inheritance from mother to offspring, and also infer paternal haplotypes.

Phylogenetic analyses were conducted using MEGA version X (45).

### *Borrelia* Infection Status and Data Selection

Infection status was determined by *flaB* real-time PCR assays with primers specific for *B. afzelii*: Fla5F 5’-CACCAGCATCACTTTCAGGA-3’ and Fla6R

5’-CTCCCTCACCAGCAAAAAGA-3’ [see (46) and (37)]. At least eight negative controls were included on each plate to check for contamination. Samples were considered positive if they had a melting temperature between 78.15–78.75°C and a Ct value corresponding to ≥ one *B. afzelii* spirochaete (47). Each sample was run in duplicates on separate plates and only samples that were positive in both replicates were scored as infected. The repeatability of infection status between replicates of the same sample has previously been estimated to 0.92 (46).

Previous analyses have shown that *B. afzelii* infection prevalence increases with age. We therefore performed our analyses on an aggregated data set where infection status and additional explanatory variables were extracted from the last capture event of each individual in the longitudinal data set. Choosing the last data point from each individual increases the time of exposure to infected tick nymphs and should therefore increase the statistical power to detect differences in *B. afzelii* infection status among hosts with different DQB-haplotypes.

None of the juveniles (<15 g) in our data selection were positive for *B. afzelii*. We therefore chose to exclude juveniles from the data set and included only two age classes (adult, ≥20 g; subadult, <20 ≥15 g) in the analyses. The absence of infection in very young individuals is likely explained by limited exposure to *B. afzelii*, rather than resistance. After 454-filtering and exclusion of juveniles, data from 527 bank voles remained.

### Statistical Analyses

Prevalence of infection was analysed by means of generalized linear models, using the HPGENSELECT procedure in SAS 9.4 (SAS Institute Inc., Cary, NC, USA) with binary error distribution. The effect of age and month were included in all models. Forward and backward model selection yielded identical conclusions.

To test for effects of specific haplotypes on infection status, we inlcuded each haplotype as a factor (coded as present/absent in each individual). We wanted to include as many haplotypes as possible since rare haplotypes are predicted to be more resistant under NFDS and chose to include all haplotypes that were found in >5% of the individuals. In total, ten DQB-haplotypes were analysed. We included all two-way interactions between host age class and DQB-haplotype in the initial statistical model. Effect sizes of effects of specific alleles were estimated as partial correlation coefficients (i.e correlations while controlling for other significant factors) using the CORR procedure in SAS 9.4.

To test the effect of MHC diversity on infection status, we tested for an effect of number of alleles within an individual. We also included the quadratic term for number of alleles in the model (to test the ‘optimality hypothesis;’ (48, 49).

To test the DAA hypothesis, we performed analyses of infection status against allelic divergence within individuals. Allelic divergence within individuals was measured as the average pairwise amino acid distance between all alleles (p-distance) within an individual using MEGA 5.0.5 (50). The genetic distance between alleles in a genotype has been shown to correlate with the combined number of bound peptides of that genotype (28). In humans, where the residues of the PBR are known, the correlation does not increase when the non-PBR is excluded (28). Therefore, genotype divergence was measured as the average pairwise p-distance of the whole DQB sequences rather than selecting the PBR inferred from mouse. The DAA hypothesis should be applicable to systems with multiple MHC loci where each allele bind a limited number of antigens (28). Besides the average pairwise p-distance between all alleles within an individual, we also included number of alleles as a factor to control for possible effects of MHC diversity on p-distance.

## Results

### Characterization of DQB Haplotypes

**Figure 1:**
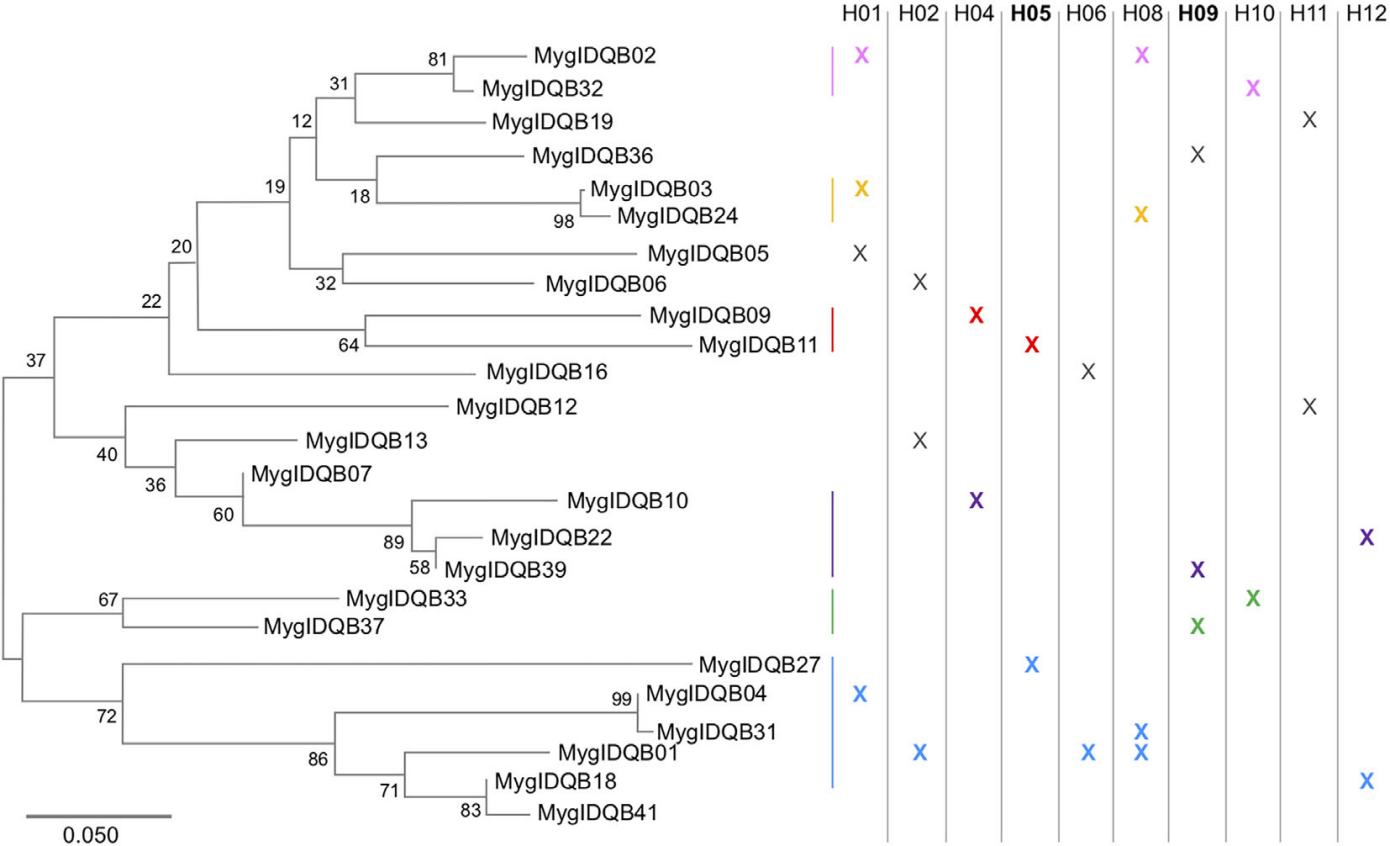
Twenty five DQB alleles occurred in >5% of the studied individuals and 23 of these alleles could be placed in ten different multi allelic haplotypes (H01, H02 etc., each occurring in >5% of the studied individuals). A phylogenetic reconstruction showed six allelic clusters with reasonable bootstrap support (boots trap values 64-98, indicated by different colours (lines and x), whereas alleles in non-supported clusters are black) and all ten DQB haplotypes were combinations of alleles found distantly distributed in the tree (ML, Kimura 2-parameter model + G, bootstrap repeats 500).

We obtained data on DQB genotypes and *B. afzelii* infection status from 527 bank voles in our study population. Altogether we found 34 different DQB alleles and each bank vole had between one and eight alleles (median=5, **Supplementary Figure 1**). Twentyfive of the 34 alleles occurred at a frequency >5%, and by analyzing patterns of co-occurrence (**Supplementary Figure 2**), we inferred that 23 alleles formed ten different haplotypes (H01, H02 etc., **Table 1**). Two alleles, MyglDQB*07 and MyglDQB*41, could not be associated with other alleles. The DQB allele MyglDQB*01 and MyglDQB*02 were found in three and two different haplotypes, respectively, but the remaining 21 alleles were only found in a single haplotype each. The patterns of co-occurence is therefore lower per haplotype for MyglDQB*01 and MyglDQB*02 (**Supplementary Figure 2**). Phylogenetic reconstruction of the 25 DQB alleles (frequency >5%) generated six reasonably supported phylogenetic clusters (bootstrap support 64-98, 2-6 alleles per cluster) and indicate that the alleles within each haplotype are relatively distant (**Figure 1**). This pattern is expected since alleles within a locus (gene) on average have higher sequence similarity than alleles belonging to different loci.

Analysis of the family data (12 litters with mother and pups), showed as expected, one maternal MHC haplotype in each pup and two maternal MHC haplotypes per litter (the average litter size is 4.8 pups in the 14 studied litters). In four litters there were two different inferred paternal haplotypes while eight litters had three to five inferred paternal haplotypes, indicating substantial promiscuity. Nine of the ten (>5%) DQB haplotypes inferred from the patterns of co-occurrence in the main data set were confirmed by analysing segregation of alleles in mother and offspring genotypes (**Supplementary Table 1**). The last of the ten (>5%) DQB haplotypes was associated with one additional allele in the family data set; this association also occurred in the main data set, but at much lower frequency than the originally inferred haplotypes. We therefore used the haplotype originally inferred from associations in the main data in the analyses below. The fact that nine of the ten haplotypes inferred from associations in the main data set matched perfectly to haplotypes in the family data set, and the tenth matched partly, shows that our approach to infer haplotypes worked. Crucially, the two haplotypes that turned out to be associated with *B. afzelii* infection (H05 and H09; see below) were both unambigously confirmed by the family data.

In line with our previous study (33), we found a maximum of eight DQB-alleles per individual, hence DQB must be at least quadruplicated. The most abundant haplotype (H01), was found in more than one third of all bank voles (N=185; **Table 1**), and contained four alleles (MyglDQB*02, *03, *04, *05) which further supports the presence of up to four DQB-loci per individual (**Table 1**). However, most haplotypes contained only two alleles, which is likely explained by gene copy number variation between individuals, though presence of null alleles, the occurrence of rare alleles which could not be linked to specific haplotypes, and sharing of alleles between loci can also contribute. Of the 527 individuals, 263 (49.9%) carried one of the ten (>5%) DQB haplotypes, 221 (41.9%) carried two haplotypes and 43 (8.16%) did not carry any of these haplotypes.

### Effects of Specific DQB Haplotypes on Infection Status

**Figure 2:**
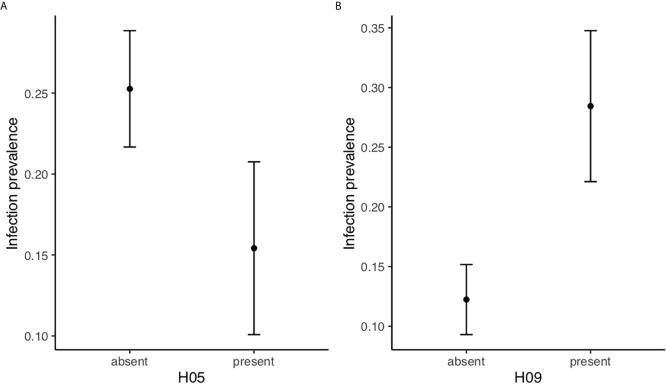
Specific MHC DQB-haplotypes are associatied with *B. afzelii* infection status. **(A)** Model estimates (LS means ± SE) of *B. afzelii* prevalence in bank voles (n=527) with haplotype H05 (n=77) is lower than for bank voles without H05. **(B)** Model estimates of *B. afzelii* prevalence in bank voles with haplotype H09 (n=44) is higher than for bank voles without H09. The model included age and month as class variables as well as H05 and H09.

We investigated if either of the ten (>5%) DQB haplotypes had an effect on infection status. One hundred and six of the 527 individuals (20.1%) were infected with *B. afzelii*. The final model included the effect of age, month and the presence/absence of the two MHC DQB haplotypes H05 and H09 (age: $\chi_{\left(1\right)}^{2}$ = 34.82, p<0.0001; month: $\chi_{\left(3\right)}^{2}$ = 14.63, p=0.0055; H05: $\chi_{\left(1\right)}^{2}$ =4.61, p=0.032; H09: $\chi_{\left(1\right)}^{2}$ = 6.81, p=0.0091, see **Supplementary Table 2** for details of model selection). All interactions and the main effect of all other haplotypes were removed in the model selection procedure. Bank voles with haplotype H05 were less often infected with *B. afzelii* than voles without this haplotype, indicating that H05 confers resistance (parial *r*=−0.087; **Figure 2A**). Conversely, bank voles with haplotype H09 were more often infected with *B. afzelii* than individuals without this haplotype, indicating that H09 confers susceptibility (partial *r*=0.12; **Figure 2B**).

Adults were infected more than three times as often as subadults in an initial model accounting for seasonal effects of month (adults: 31.9%; subadults: 9.7%; $\chi_{\left(1\right)}^{2}$ = 38.85, p<0.0001). There was no interaction between age and month $\chi_{\left(3\right)}^{2}$ = 3.46, p=0.33).

### Effects of MHC Diversity and Allelic Divergence Within Individuals on *Borrelia* Infection Status

There was no effect of MHC diversity on infection status, (number of alleles: χ2(1)=1.54, p=0.21, (number of alleles)^2^: χ2(1)=0.56, p =0.45).

We found no significant relationship between allelic divergence within individuals [average pairwise amino acid distance between all alleles (p-distance) within an individual] and infection status [p-distance: χ2(1)=0.00, p=0.98], and no interaction between allelic divergence within individuals and number of alleles [p-distance × number of alleles: χ2(1)=0.03, p=0.87].

### The Effect of Specific Haplotypes on Allelic Divergence Within Individuals

**Figure 3:**
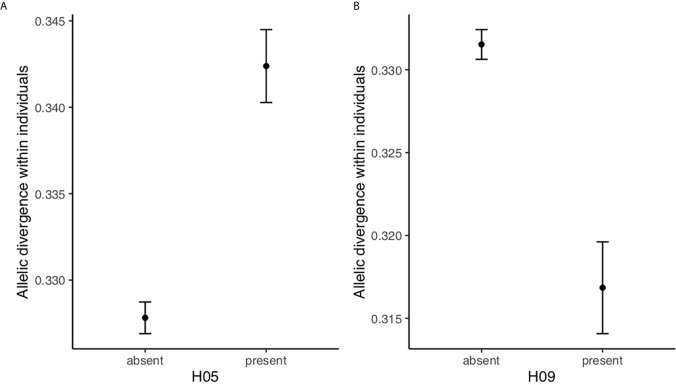
Allelic divergence within individuals (LS means ± SE) for heterozygous bank voles is higher in bank voles with the resistance haplotype H05 and lower in bank voles with the susceptibilioty haplotype H09 **(A)** with and without haplotype H05, **(B)** with and without haplotype H09.

Finally, we tested if the resistance haplotype H05 and the susceptibility haplotype H09 had an effect on allelic divergence within individuals. These models were run on a data set including only heterozygous individuals (i.e. individuals that either carried two different (>5%) DQB haplotypes (N=221) or one (>5%) DQB haplotype plus at least one more allele (N=189); in total N=410). We only included heterozygous individuals because we are interested in testing how the resistance and susceptibility haplotypes (H05 and H09, respectively) affect the allelic divergence within individuals rather than the allelic divergence within each haplotype. In other words, we want to test if the allelic divergence within individuals carrying resistance/susceptibilty haplotypes differs from other individuals. Individuals with the resistant haplotype H05 had more divergent genotypes than individuals without this haplotype (F1, 408 = 40.1, p <0.0001; **Figure 3A**), indicating that individuals carrying haplotype H05 have a broader peptide binding repertoire. On the contrary, individuals with the susceptible haplotype H09 had less divergent genotypes than individuals without this haplotype (F1, 408 = 25.430.63, p=<0.0001; **Figure 3B**), indicating that individuals carrying haplotype H09 have a more narrow peptide binding repertoire.

## Discussion

We found significant effects of two out of ten bank vole DQB haplotypes on *B. afzelii* infection status. The haplotype H05 was associated with lower prevalence of infection (resistance) while the haplotype H09 was associated with higher prevalence of infection (susceptibility). Moreover, heterozygous individuals with the resistance haplotype H05 had higher allelic divergence within individual than heterozygous individuals without the H05 haplotype, which should enable a broader peptide-binding repertoire and consequently improve the ability to eliminate pathogens. Conversely, heterozygous individuals carrying the susceptibility haplotype H09 had lower allelic divergence within individual than other individuals, which should result in a more narrow peptide-binding repertoire and restrict the ability to eliminate pathogens. Associations between host genotype and prevalence of infection could either be a result of host genotypes affecting the pathogen’s possibility of establishing an infection in the host, or the ability of the host to clear an established infection.

The pattern found in the present study, where specific haplotypes, H05 and H09, were associated with resistance and susceptibility, respectively, to a specific pathogen suggests that NFDS and/or FS are involved in maintaining DQB allelic diversity. The finding that individuals carrying the resistance haplotype H05 also have higher genotypic divergence is consistent with the scenario envisaged by (27), where NFDS favours alleles that are relatively different from other alleles in the population (note that only heterozygous individuals were included in these analyses). Haplotype H05 have two different DQB alleles (Mygl-DQB*11 and Mygl-DQB*27), and interestingly one of the alleles, Mygl-DQB*27, is distant to the other DQB alleles according to the phylogenetic reconstruction, i.e. it suggests that Mygl-DQB*27 is rather different from other alleles in the population. As pointed out in the introduction, the scenario envisaged by (27) does not imply selection for allelic divergence within individuals *per se* (DAA). Instead divergent alleles are selected because they provide resistance. Selection for divergent alleles at the population level may in this way result in high allelic divergence within individuals for individuals carrying resistant alleles under NFDS.

According to theory, very low and very high MHC diversity is selected against (48, 49). This is because individuals with very low MHC diversity, few MHC alleles, have a limited ability to present antigens while individuals with very high MHC diversity loose large proportions of their T-cells during the maturation of the adaptive immune system, giving rise to potential gaps in their T-cell repertoires. Thus, there is a trade-off resulting in that individuals with an optimal MHC diversity are selected for (48, 49). Bank voles in our study population have between one and eight MHC alleles, hence we have a suitable data set to investigate the effect of MHC diversity, though we did not find an overall effect of MHC diversity on infection status. Furthermore, we did not find an overall effect of allelic divergence within individuals (DAA) on infection status, but a handful of previous studies have found that DAA plays a significant role in relation to resistance/susceptibility to infection, or for survival (16, 30–32, 51). We study DQB in a multi-locus system and would like to acknowledge that effects of DAA might not be readily detectable when individual loci cannot be analysed seperately. Moreover, we only analyzed effects of MHC diversity or DAA on resistance to one pathogen, and it is likely MHC diversity and DAA are more important when resistance to infection by multiple parasites is considered.

In wild mammals, both positive and negative associations between specific MHC class II alleles and infectious disease have been reported in the case of helminth infections (13, 38, 52–56), as well as protozoa (10, 56, 57) and bacteria (10), whereas MHC class II had no detectable effect in case of bacterial infections with *Bartonella* in water voles (9), *Mycoplasma* in bank voles (14), or *Salmonella* and *Campylobacter* in badgers (10). In our study, haplotype H05 was associated with reduced prevalence of *B. afzelii*, whereas haplotype H09 was associated with increased prevalence. A specific MHC allele/haplotype can be associated with resistance to a particular pathogen simply by being able to bind and present peptides from the pathogen, so called ‘determinant selection’ (58). However, it is not immediately clear how a specific allele or haplotype can be associated with susceptibility. One explanation for MHC-susceptibility associations is that some peptide-MHCs fail to activate T-cells (59–62). MHC-molecules that bind antigens that are similar to host self (autologous antigens) are associated with non-responsiveness of T-cells (63, 64) and consequently with susceptibility to disease (59, 61). It has been suggested that parasites that cause persistent infections, might evolve proteins that are similar to host self-proteins (molecular mimicry (65);). Hosts must ignore such antigens because they can potentially induce autoimmune disorders (59, 66). It is known that another *Borrelia* species, *B. burgdorferi* sensu stricto, is associated with the onset of an inflammatory disease in humans (arthritis), possibly caused by a *Borrelia* protein being similar to a human protein (67–69). An alternative explanation for MHC-susceptibility is that bank voles with the H09 haplotype are in fact relatively resistant and are able to keep the infection at lower intensity but not clear it [i.e. ‘quantitative resistance’ (20, 70)];, leading to increased survival, whereas individuals lacking this allele succumb to high infection intensities (20). Additional potential explanations for MHC-susceptibility haplotypes could be that a pathogen recently has evolved away from what used to be an MHC-resistance haplotype and individuals with this haplotype therefore are frequent in the population though fail to detect the pathogen (71).

To conclude, we found that bank vole MHC class II DQB haplotypes can be associated with both resistance and susceptibility to natural infections with *B. afzelii.* Moreover, heterozygote individuals carrying the resistance haplotype had higher allelic divergence within individuals than individuals without it, while the reverse was true for individuals carrying the susceptibility haplotype. This pattern of a higher allelic divergence in individuals with a resistance haplotype is consistent with NFDS favouring divergent alleles (27).

## Data Availability Statement

The new DQB-alleles and the datasets used in this study can be found in online repositories. GenBank accession numbers of DQB alleles: MZ359152, MZ359153, MZ359154, MZ468899 and MZ468900. Population data sets: Scherman, Kristin; Råberg, Lars; Westerdahl, Helena (2021), Borrelia infection in bank voles Myodes glareolus is associated with specific DQB haplotypes which affect allelic divergence within individuals, Dryad, Dataset, https://doi.org/10.5061/dryad.x69p8czjj.

## Ethics Statement

The animal study was reviewed and approved by Malmö/Lund ethical board for animal experiments (permits M101-06 and M141-10).

## Author Contributions

KS, LR, and HW designed the study and wrote the manuscript. KS and LR did field work and perfomed data analyses, and KS did the lab work. All authors contributed to the article and approved the submitted version.

## Funding

The project was funded by the Swedish Research Council (Grants 621-2006-2876 and 621-2006-4551 to HW and LR, respectively), by Carl Tryggers Stiftelse för Vetenskaplig Forskning to HW, by Crafoordska stiftelsen to HW and LR, and by Stiftelsen Lunds Djurskyddsfond to KS.

## Conflict of Interest

The authors declare that the research was conducted in the absence of any commercial or financial relationships that could be construed as a potential conflict of interest.

## Publisher’s Note

All claims expressed in this article are solely those of the authors and do not necessarily represent those of their affiliated organizations, or those of the publisher, the editors and the reviewers. Any product that may be evaluated in this article, or claim that may be made by its manufacturer, is not guaranteed or endorsed by the publisher.
