# Supplementary material for: Borrelia Infection in Bank Voles Myodes glareolus Is Associated With Specific DQB Haplotypes Which Affect Allelic Divergence Within Individuals
Source: Front Immunol. 2021 Jul 26;12:703025. doi: 10.3389/fimmu.2021.703025 (PMC8350566; doi:10.3389/fimmu.2021.703025)
Supplement: Supplementary file 1 [file Presentation_1.pdf]

Borrelia infection in bank voles *Myodes glareolus* is associated with specific DQB haplotypes which affect allelic divergence within individuals.

Supplementary Material

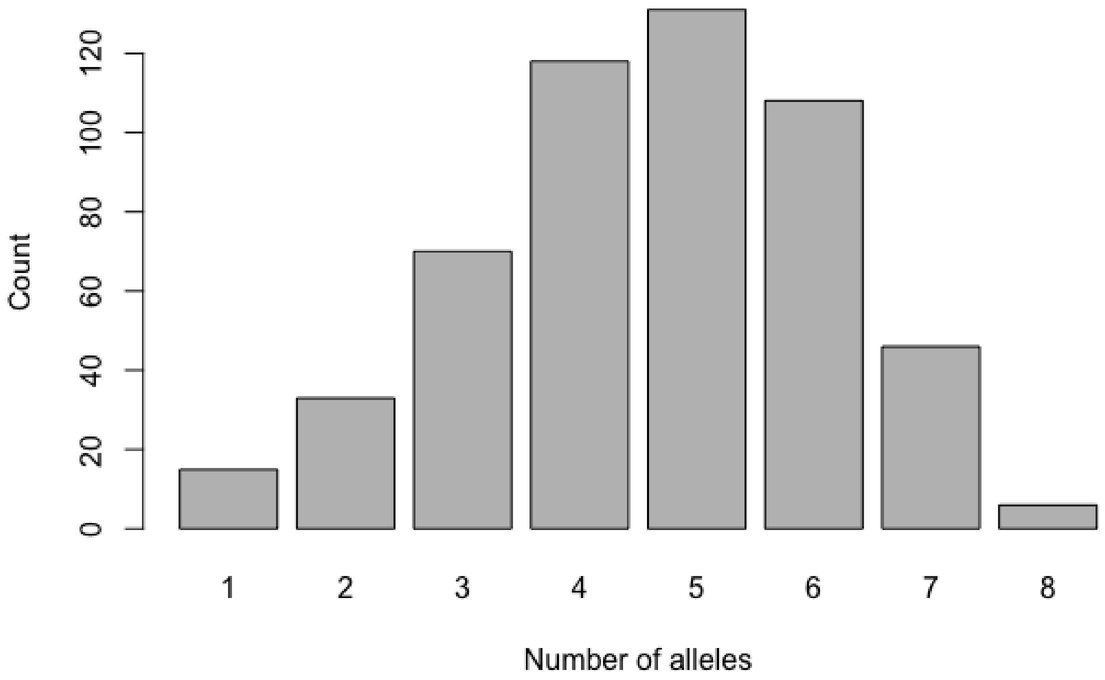

Figure S1. Frequency distribution of number of DQB alleles per individual bank vole.

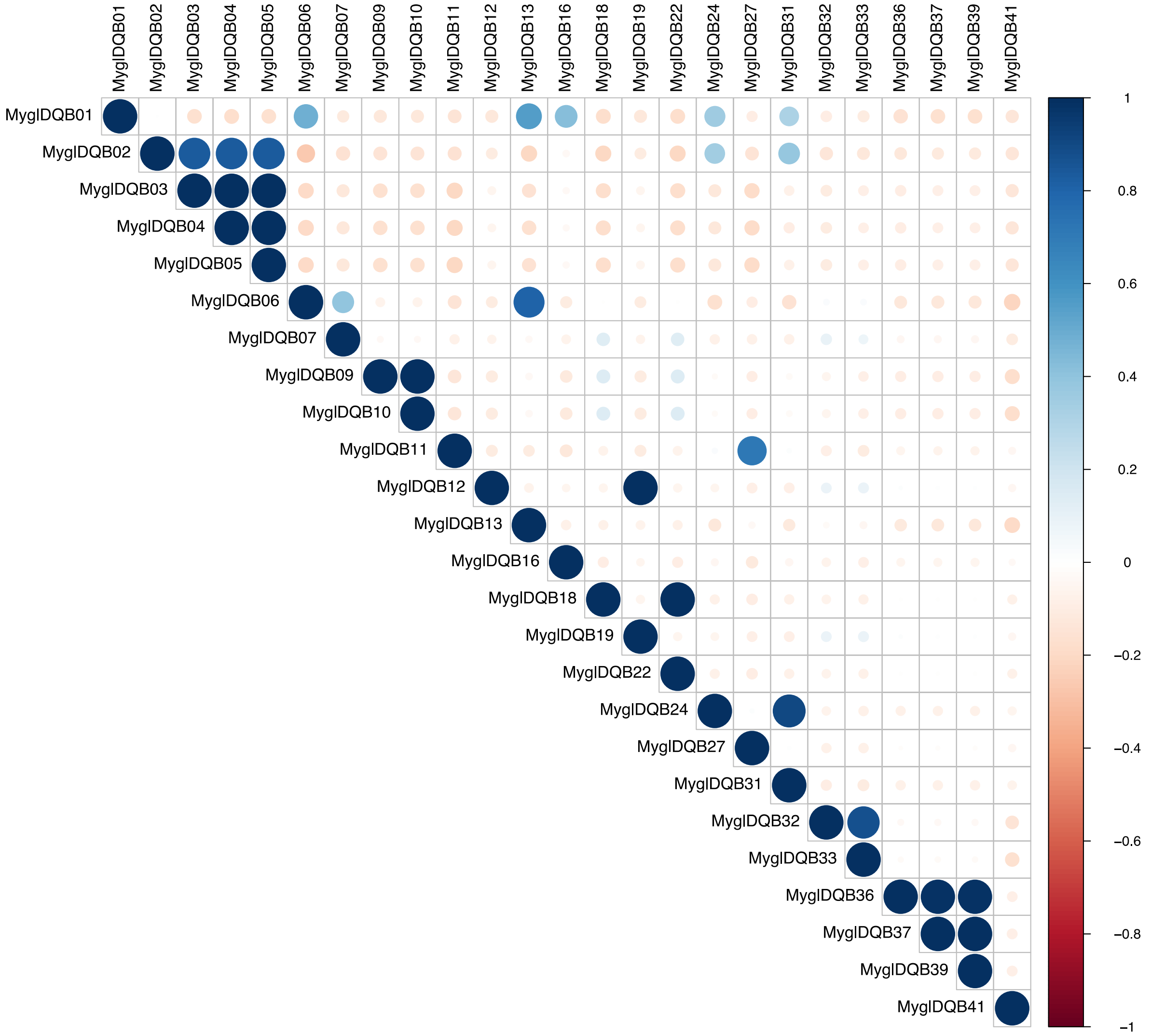

Figure S2. Correlations among DQB alleles that occurred in >5% of bank voles.

Table S1. Allelic composition of the ten haplotypes (Haplotype, H01, H02 etc.) that occurred in >5% of the studied individuals (DQB alleles in haplotypes based on association in main data set). These ten haplotypes were also found in an independent data set with DQB genotypes from 12 families, with known mother-offspring relations, and they were verified using mother-offspring data and/or using the inferred parental haplotypes.

**Haplotype**	**DQB alleles in haplotypes based on association in main data set**	**Verified by comparison of mother-offspring in family data**	**Inferred parental haplotype in family data**	**Comment**
**H01**	*02*03*04*05	Yes	Yes	
**H02**	*01*06*13	Yes	Yes	
**H04**	*09*10	Yes		
**H05**	*11*27	Yes	Yes	
**H06**	*01*16		Yes	*16 not found in any of the mothers, but *01 and *16 together in one offspring
**H08**	*01*02*24*31	Yes	Yes	
**H09**	*36*37*39		Yes	None of these alleles found in any of the 12 mothers, but perfectly associated in five offspring from three different broods.
**H10**	*32*33	Yes		
**H11**	*12*19		Yes	*12 and *19 not found in any of the mothers, but perfectly associated in four offspring from four different broods.
**H12**	*18*22	(Yes)		*18*22 was also associated with *20 in the mother-offspring in family data

Table S2. Results of forward model selection analysis of factors affecting infection status with *Borrelia afzelii.* Explanatory variables included month of capture, age, presence/absence of 10 DQB haplotypes and their interactions with age. Forward and backward model selection yielded the same conclusions. Final model χ^2/df=1.03.

Step	Factor	χ^2	*P*	AIC
1	Initial Model			531.08
2	Age	40.19	<0.0001	491.79
3	Month	14.62	0.0055	483.91
4	H09	6.86	0.0088	465.63
5	H05	4.79	0.0286	476.34
